# Circulating Vitamin D levels status and clinical prognostic indices in COVID-19 patients

**DOI:** 10.1186/s12931-021-01666-3

**Published:** 2021-03-03

**Authors:** Alberto Ricci, Alessandra Pagliuca, Michela D’Ascanio, Marta Innammorato, Claudia De Vitis, Rita Mancini, Simonetta Giovagnoli, Francesco Facchiano, Bruno Sposato, Paolo Anibaldi, Adriano Marcolongo, Chiara De Dominicis, Andrea Laghi, Emanuele Muscogiuri, Salvatore Sciacchitano

**Affiliations:** 1 Respiratory Unit, Sant’Andrea Hospital, Sapienza University of Rome, Via di grottarossa, 1035 Rome, Italy; 2 Sant’Andrea Hospital, Rome, Italy; 3 Laboratory of Biomedical Research, Niccolò Cusano University Foundation, Via Don Carlo Gnocchi 3, 00166 Rome, Italy; 4 Department of Oncology and Molecular Medicine, Istituto Superiore Di Sanità, Rome, Italy; 5 Respiratory Unit, Misericordia Hospital, Grosseto, Italy

## Abstract

**Background:**

Several immune mechanisms activate in COVID-19 pathogenesis. Usually, coronavirus infection is characterized by dysregulated host immune responses, interleukine-6 increase, hyper-activation of cytotoxic CD8 T lymphocytes. Interestingly, Vitamin D deficiency has been often associated with altered immune responses and infections. In the present study, we evaluated Vitamin D plasma levels in patients affected with different lung involvement during COVID-19 infection.

**Methods:**

Lymphocyte phenotypes were assessed by flow cytometry. Thoracic CT scan involvement was obtained by an image analysis program.

**Results:**

Vitamin D levels were deficient in (80%) of patients, insufficient in (6.5%) and normal in (13.5%). Patients with very low Vitamin D plasma levels had more elevated D-Dimer values, a more elevated B lymphocyte cell count, a reduction of CD8 + T lymphocytes with a low CD4/CD8 ratio, more compromised clinical findings (measured by LIPI and SOFA scores) and thoracic CT scan involvement.

**Conclusions:**

Vitamin D deficiency is associated with compromised inflammatory responses and higher pulmonary involvement in COVID-19 affected patients. Vitamin D assessment, during COVID-19 infection, could be a useful analysis for possible therapeutic interventions.

*Trial registration:* 'retrospectively registered'.

## Background

COVID-19 infection is still an open challenge to date. Although the clinical features following the penetration of the virus into our respiratory system are known, the pathobiology and the mechanisms that regulate this entry and the reasons behind the multivarious clinical pictures observed are still unknown. Unfortunately, about 20% of infected patients developed a severe respiratory disease characterized by diffuse pulmonary infiltrates and damage of alveolar type II pneumocytes, which undergoes apoptosis and death [1]. The involved alveolar units appear to be peripheral and subpleural. Furthermore, a viral driven hyperinflammation has been reported [2]. An early overproduction of pro-inflammatory cytokines has been described and defined cytokine storm [2, 3]. Among them, elevated IL-6 plasma levels were included as predictor of mortality [4]. Recent observations have shown that Vitamin D (VitD) is not a mere micronutrient involved in calcium metabolism and bone healthy but it also plays an important role as a pluripotent hormone in several immunological mechanisms [5].

It is known that enzymes catalyzing its activation and the VitD receptors (VDR), that mediates the actions of the vitamin itself, are widely distributed on the whole cell bodies and in particular in pulmonary alveolar epithelium and immune system. Although the in-vivo effects of VitD are not completely understood, a number of observations underline the VitD role in lung infection and in lung diseases’ development [5, 6]. VitD insufficiency has been related to viral infections of the lower respiratory tract [7] and to exacerbation in chronic obstructive lung diseases and asthma [5, 6].

Normally, VitD ingested or endogenously produced is hydroxylated to 25-hydroxy VitD (25HOD) by the liver. This metabolite is measured to assess VitD status. Its hormonal activity is reached after renal 1a-hydroxilase’s activation to 1,25OH2D. Without any supplementation, VitD status is strictly dependent by endogenous production that may be influenced by genetic variants of VitD-binding proteins, seasons, latitude, skin and lifestyle [8, 9]. More interestingly, studies concerning VitD effects on human adaptive immune responses, demonstrated the expression of the nuclear VitD receptor and VitD activating enzymes within immune cells [10]. In both activated T and B lymphocytes an up-regulation of VitD receptors was demonstrated [10, 11]. Recent studies underline an indirect, through helper T lymphocytes and direct role of VitD on B lymphocytes homeostasis. T lymphocytes are important targets of the immunomodulatory effects of VitD.

Notably, they influence the secretion of a variety of proinflammatory cytokines [12]. Th1 lymphocytes (IL-2, INF-gamma, TNF-alpha) and VitD plasma levels are inversely related to plasma levels of IL-6 [13]. VitD down-regulates IL-6 mRNA levels [13]. On the other hand, IL-6 synthesis has been correlated with immune cell differentiation and maturation, and cytokine production [14]. In T lymphocytes cultures, VitD seems to facilitate the development of a tolerogenic phenotype and the increase of genes involved as regulatory T lymphocytes (Tregs) [15].

In subject with VitD deficiency, its supplementation is able to reduce the risk to develop different viral infections [16]. Furthermore, subjects with low levels of VitD at the time of COVID-19 testing were at higher risk to be positive for COVID-19 compared to those subjects with sufficient VitD status [17].

Therefore, our study aimed to assess whether VitD deficiency was as a risk factor to develop more severe clinical pictures and more serious lung involvement in patients suffering from coronavirus infection admitted to our hospital during COVID-19 pandemic.

## Materials and methods

### Patients

52 hospitalized patients affected with Covid-19 infection and with different degree of lung involvement, diagnosed by nasopharyngeal and oropharyngeal swabs (One-step RT-PCR Kit Qiagen, detection kit, Milan Italy) (TaqMan Fast Virus 1-Step Master Mix, Thermo Fisher, Milan, Italy), positive for the presence of the SARS-CoV-2 virus were enrolled. The research has been conducted at the Sant’Andrea Hospital, designated by the Regione Lazio as one of the COVID-19 referral Hospitals in Rome, Italy. No signs of malnutrition were reported. Comorbidities and patients’ characteristics were indicated in Table 1.

**Table 1:** Patients characteristics

RISK FACTORS	GROUP 1 (VitD < 10 ng/mL) (n = 22)	GROUP 2 (VitD ≥ 10 ng/mL) (n = 30)	P value
Sex (M/F)	9/13	16/14	NS
Age(mean ± SD)	77.5 ± 16	68.9 ± 18	NS
Co-morbidities (%) None 1 > 1	9,1 27.3 63.6	23.3 16.7 60	0.01 0.05 NS
Hypertension(%)	45.5	40	NS
Obesity(%)	11.2	30	0.05
Chronic Renal failure(%)	4	3	NS
COPD(%)	9,1	10	NS

Data are presented as mean ± standard deviation, or number (%). The chi-square test or Fisher’s exact test was used to examine the data

Exclusion criterions were recent cardiovascular accidents, diagnosis of cancer, autoimmune diseases. None of the patients enrolled in this study have had BCG vaccination, in Italy anti-tuberculosis vaccine prophylaxis is mandatory only for healthcare professionals exposed to a high risk of contagion and for those who have clinical contraindications to the use of anti-tuberculosis specific drugs.

In all patients, VitD serum levels were dosed during the acute phase of the disease. The dosage of the VitD was performed at the time of admission into the hospital, before starting any kind of therapy. At the same time, different markers of inflammation (high-sensitive C Reactive Protein hs-CRP, Procalcitonin PCT), cellular damage (hypersensitive troponin I, creatin kinase miocardial band, lactate deidrogenase) and coagulation (prothrombin time, fibrinogen and D-dimer) were assessed.

Flow cytometry was performed in the whole patients’ cohort by the automated AQUIOS CL® "load & go" flow cytometer (Beckman Coulter, Life Sciences Division, Indianapolis, USA), to define peripheral lymphocyte cell type count.

The interleukin-6 (IL-6) was measured using a dedicated kit (IL-6 Human SimpleStep Elisa Kit, Thermo Fisher, Milan, Italy).

### CT acquisition

Chest CT scans were performed on all patients with COVID-19 infection using two 16-row multi-slice CT scanners. Patients were placed in a supine position and advanced head, continuous spiral scanning was performed from the lung top to the lung bottom. For CT acquisition, the tube voltage was 120 kVp with automatic tube current modulation and pitch 0.99–1.45 mm. From the raw data, CT images were reconstructed on a matrix of 512 × 512 and a field of view 350 × 350 mm as axial images (section thickness of 10 mm) The thickness of the axial reconstruction layer was 1.0 mm, the window width was 1000–2000 HU, and the window level was 700–500 HU. Quantitative results were individually assessed by two senior radiologists, and discussions were used to resolve differences in data interpretation.

### Quantification of lung CT lesions by PII

The results of lung computed tomography (CT) scans were quantified using the pulmonary inflammation index (PII). This modified semi-quantitative scoring system (Lung Quantitative Software -Siemens) was used to quantitatively assess pulmonary involvement in all patients according to lung lesion distribution and size. A total of 20 lung segments (in both left and right lungs) were assessed. The lesion size score was based on the occupation major or minor than 50% of the lung segment, by the lesion. The score was 1 for ≥ 50% involvement and zero for < 50%, with a maximum total score of 20. Higher the value, the more severe inflammatory load. PII = lesion distribution score + lesion size score/total score (40 points) × 100%.

We arbitrarily divided patients with CT scan evaluation into two groups on the basis of lung involvement: TSS ≤ 7 and TSS > 7.

### SOFA score

The Sequential Organ Failure Assessment (SOFA) score is a mortality prediction score based on the degree of dysfunction of six organ systems (Respiratory system, Nervous system, Cardiovascular system, Liver, Coagulation, Kidneys). It depends on the following variables: PaO2/FiO2, Platelets count in × 10 ^3/µL, Glasgow Coma Scale from grade 0 to + 4, bilirubin value measured in mg/dL (μmol/L), mean arterial pressure measured in mmHg, creatinine, mg/dL (μmol/L) [18]. Epidemiological, anamnestic and clinical data regarding patients are listed in Table 1 (Table 1).

Analyzing SOFA score results, we arbitrarily classified patients into two groups. The first group resulted in SOFA < 2, the latter in SOFA > 2.

### Lung immune prognostic index score

The Lung Immune Prognosis Index (LIPI) score was obtained combining baseline dNLR and LDH. It is usually considered as a prognostic value, irrespective of treatment modality, in several human malignancies. By LIPI SCORE we categorized 3 groups: good (dNLR < 3 + LDH 3 + LDH < upper limit of normal (ULN), intermediate (dNLR > 3 or LDH > ULN), poor risk (dNLR > 3 + LDH > ULN) [19]. Analyzing LIPI score, we identified two main groups: the first had LIPI 0–1 values, the second had LIPI = 2.

### Statistical analysis

Continuous variables (quantitative) are described by mean and standard deviation (SD) while categorical variables by frequency or percentage. The comparison of quantitative variables (inflammatory markers, markers of cellular damage, and coagulation) between groups was performed using the Student's t test and by the Mann‐Whitney‐Wilcoxon text for independent samples. The chi-square test or Fisher’s exact test was used to examine categorical data (SOFA; LIPI and CT score). The correlation between VitD and other parameters was assessed using Spearman’s correlation test.

Descriptive statistical analysis was performed on raw data where applicable. Results were expressed as means ± SD. A two-tailed P value of 0.05 or less was used as a criterion to indicate statistical significance. NS = not significant. Data were statistically analyzed using the GraphPad software (version) (GraphPad Software, San Diego, CA).

### Ethical approval

A written informed consent was obtained by participants to the study. The study was approved by our Institutional Ethical Committee (University La Sapienza of Rome, Italy) (Prot.# 52SA_2020, RIF. CE 5773_2020), on the basis that it complied with the declaration of Helsinki and that the protocol followed existing good clinical practice guidelines.

## Results

Fifty-two patients affected with COVID-19 infection with lung involvement were enrolled in the study (27 female and 25 male, the median age was 68,4 years, ranging from 29 to 94 years) (Patient’s characteristics reported in Table 1). The mean age of the patients with very low VitD plasma levels (under 10 ng/ml) and patients with VitD plasma levels over 10 was 77.5 + 16 and 68,9 + 18 respectively. Therefore, patients were classified into two subgroups, according to VitD plasma levels. The former “under 10 ng/mL”, named Group 1 (mean value 5.65 + 2.43 ng/mL), and the latter, “over 10 ng/mL” named Group 2 (21,54 + 8,81 ng/dL). Data regarding flow cytometry are listed in Table 2.

**Table 2:** Representative hematological findings in patients belonging to Group 1 and 2

	GROUP 1	GROUP 2	*P* value
CD8 + (absolute count)	147 ± 106	292 ± 185	0.01
CD4 + /CD8 +	2.84 ± 1.55	1.29 ± 0.37	0.001
Lymphocytes (10^3^/mm^3^)	1.27 ± 0.79	1.99 ± 0.89	0.03
Neutrophils (10^3^/mm3)	6.4 ± 1.7	4.40 ± 3.08	NS
NLR	7.04 ± 3.86	3.11 ± 2.2	0.002
dNLR	5.8 ± 2.4	3.2 ± 2.6	0.001
IL6 (pg/ml)	85.2 + 78.01	39.6 + 17.87	0.009
D-Dimer (ng/mL)	1410 + 995	519 + 930	0.02

Data are presented as mean ± standard deviation, Comparison of quantitative variables (inflammatory markers, markers of cellular damage, and coagulation) between groups was performed using the Student's t test and by the Mann‐Whitney‐Wilcoxon text for independent samples. *IL-6* interleukin 6, *NLR* Neutrophil/Lymphocyte, *dNLR* derived neutrophil to lymphocyte ratio (dNLR; absolute neutrophil count/[white blood cell concentration − absolute neutrophil count])

**Fig. 1:**
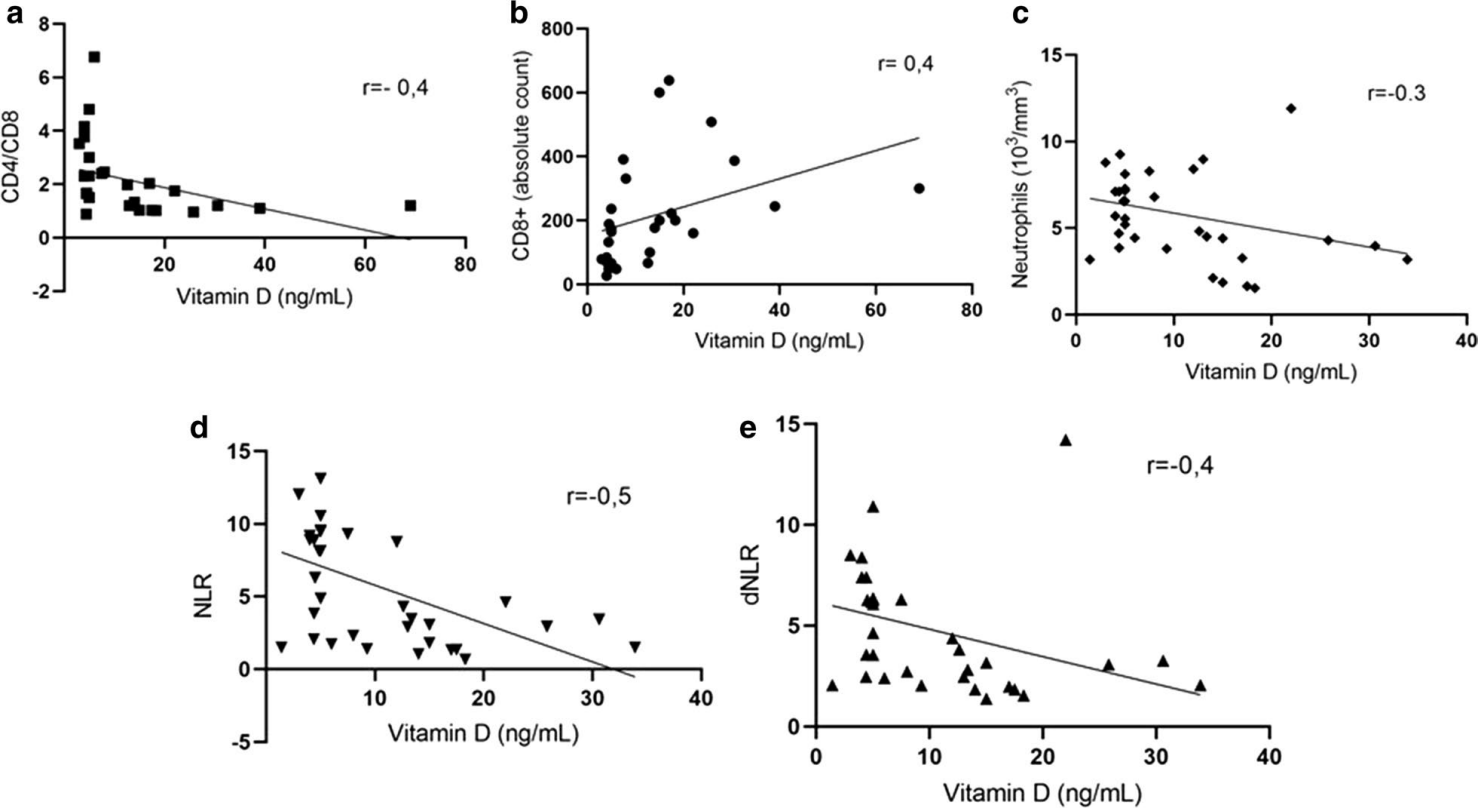
We detected an inverse correlation between VitD levels and CD4/CD8, Neutrophil cell count, NLR and dNLR. Contrarily, a direct correlation was detected comparing VitD levels and CD8 + cell count. The correlation between VitD levels and other parameters was assessed using Spearman’s correlation test

**Fig. 2:**
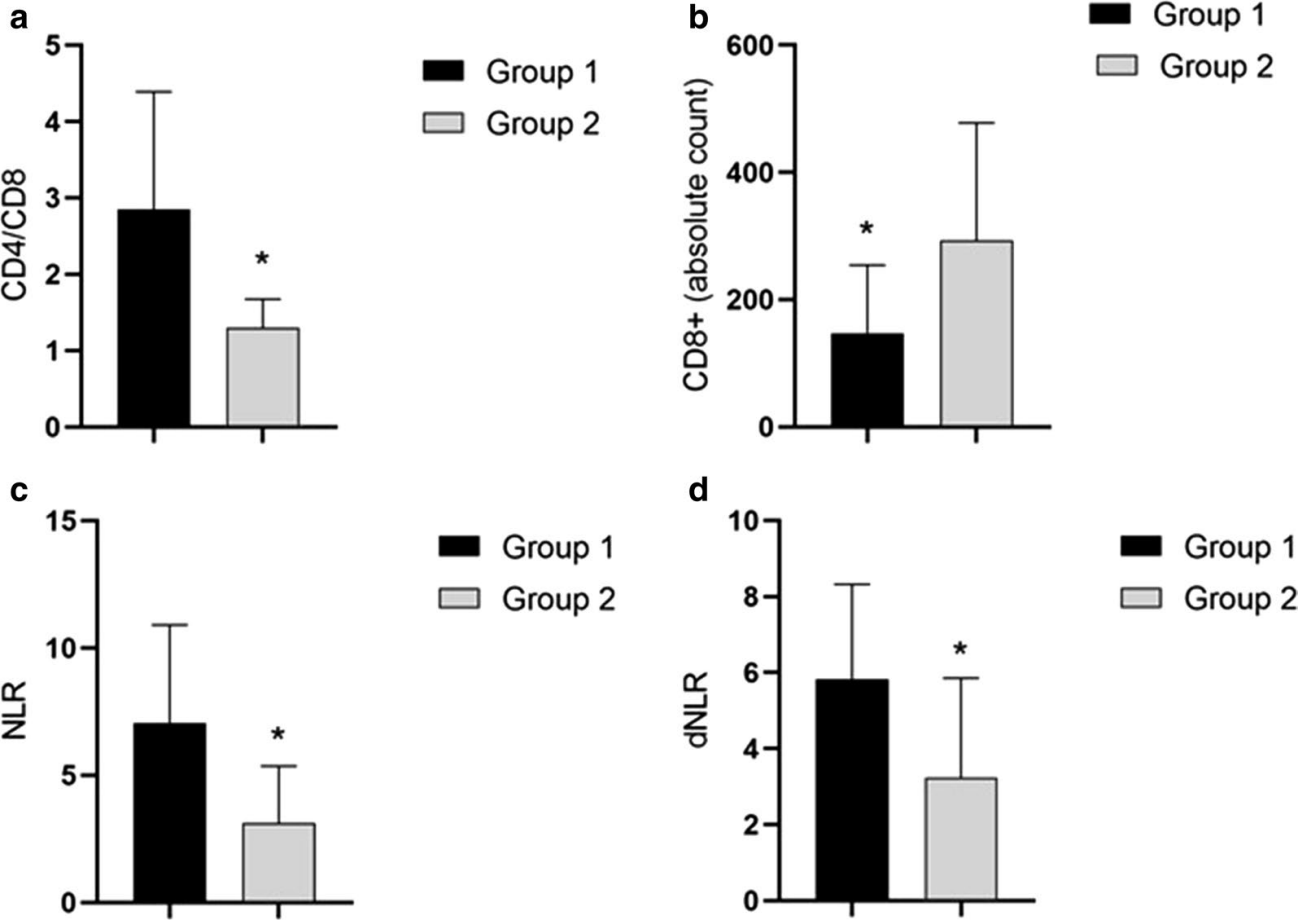
The histograms indicate that patients belonging to Group 1 had a statistically significant difference with Group2. The **a** indicate that patients in Group 1 with low Vit D levels had more elevated CD4/CD8 ratio (**a**), NLR (**c**) and dNLR (**d**). Contrarily, CD8 + cell count was lower in Group 1 than Group 2 (**b**). The comparison of quantitative variables between groups was performed using the Student's t test and by the Mann‐Whitney‐Wilcoxon text for independent samples

Although any significant difference in lymphocyte cell count has been found between the two groups, by flow cytometry, a moderate correlation was demonstrated comparing VitD plasma levels to TCD8 + cytotoxic lymphocytes and CD4 + /CD8 + ratio in both groups (Fig. 1a, b). In particular, a statistically lower TCD8 + cell count was observed in Group 1 in comparison to Group 2 and (Fig. 2b), consequently, CD4 + /CD8 + ratio resulted increased (Fig. 2a). Furthermore, a robust correlation was identified comparing VitD and Neutrophils cell count, Neutrophils and Lymphocytes ratio (NLR) and derived Neutrophils/ Leucocytes-Neutrophils ratio (dNLR) (Fig. 1c–e). A statistically significant increase in NLR, Neutrophils cell count and dNLR was detected in Group 1, if compared to Group 2 (Table 2 and Fig. 2c, d).

Assessing IL-6 plasma levels, the key element in cytokines’ inflammatory scenario, high variability of values was found among patients. However, a significant difference was documented in Group 1, despite Group 2 (Table 2).

In patients belonging to Group 1, D-Dimer plasma levels were statistically increased in comparison to Group 2 (Table 2), and a statistically significant difference was found.

A correspondence between VitD and platelets count has been documented, although not statistically significant. Pro-calcitonin levels were also investigated and no difference between the two groups was demonstrated.

Other inflammatory markers (hs-CRP), cellular damage markers (such as hypersensitive Troponin, creatin kinase myocardial band, lactate dehydrogenase), coagulation markers (fibrinogen and prothrombin time) studied did not demonstrated statistically significant differences between the two groups.

**Fig. 3:**
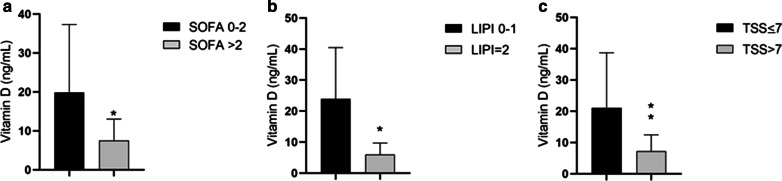
The histograms highlighted that patients that display lower levels of Vitamin D had lower SOFA (**a**), LIPI (**b**) scores and TSS (**c**) values. The chi-square test or Fisher’s exact test was used to examine categorical data (SOFA; LIPI and CT score). *p < 0.03. **p < 0.01

We detected lower SOFA score, LIPI score and TSS values in patients with higher VitD levels. These patients were mainly included in the Group 2. Obviously, patients with higher SOFA and LIPI score, and higher TSS values displayed lower VitD levels, belonging to the Group 1 (Fig. 3a–c). Moreover, assessing LIPI score we documented that 62.5% were LIPI 2 (poor prognosis), 33.4% were LIPI 1 (intermediate prognosis), and 4.1% were LIPI 0 (good prognosis).

Although the number of patients was not particularly high, such as to allow a clear statistical evaluation, Group 1 showed a higher mortality rate than Group 2. In fact, three patient died in Group 1 but none in Group 2.

## Discussion

The present study compared, for the first time, VitD plasma levels with different health related scores, inflammatory markers, markers of cellular damage and coagulation and radiological findings during COVID-19 illness. Interestingly, patients with low VitD plasma levels had compromised biochemical and clinical findings reflected by the profound immunological involvement. Vitamin D insufficiency and deficiency were common in COVID-19 affected patients. About 8% of the study cohort had normal VitD plasma levels. Patients with more severe COVID-19 disease had lower Vit D plasma levels regardless of age.

VitD plasma levels are the most accurate markers for defining VitD state. 25(OH)D is the major form of circulating of VitD and it is the one measured. In general, in population VitD levels lower than 20 ng/mL (50 nmol/L) are commonly considered as deficiency status. Moreover, levels ranging 20 to 29 ng/mL (52–72 nmol/L) should define insufficiency, while levels above 30 (75 nmol/L) sufficiency [20]. Moreover, these levels are strictly related to VitD effects on bone metabolism, while VitD levels useful for investigation of other aspects of human health are under investigation [10, 20]. For a long time, 10 ng/ml has been considered the VitD level for defining deficiency [21, 22]. Although the Endocrine Society has defined 20 ng/ml VitD plasma levels, in terms of 25(OH)D, as the threshold to define the deficiency status [23] there are insufficient evidences to clarify the optimal plasma VitD concentration necessary for the global well-being [23–25].

From a general point of view, VitD activity also seems essential in the regulation of oxidative stress and survival mechanisms [26]. Furthermore, a broad number of studies suggests the role of VitD not only as a simple micronutrient, linked to calcium homeostasis, but as a pluripotent hormone which has extensive immuno-modulatory functions [27]. The respiratory alveolar epithelium represents the first line of defense able to counteract and prevent the entry of inhaled pathogens. It represents one of the main actor of the innate immunity including alveolar macrophages and dendritic cells. If stimulated, these cells activate a variety of intracellular signaling pathways for specific antimicrobial defenses, release of inflammatory mediators and adaptive immune responses [28]. Adaptive immune response is strictly related to the ability of T and B lymphocytes to secrete cytokines and produce immunoglobulins respectively [29]. VitD deficiency has been correlated with increasing levels of IL-6[30], while VitD supplementation down regulates IL-6 levels in several studies [31]. IL-6 is elevated in COVID-19 patients with severe disease and it is also considered a relevant prognostic marker. It has been reported that mortality is higher in patients with elevated levels of IL-6. Therefore, IL-6 and IL-6R are receiving more attention as potential therapeutic targets for the treatment of COVID-19. In our COVID-19 infected patient group we have documented elevated IL-6 levels, upper the normal limits, in almost all the patients. Unfortunately, large variability among them was documented. Therefore, no correlation was detectable between VitD and IL-6 values in this cohort of patients. Moreover, in patients with very low VitD levels, the IL-6 values was slightly, but not significantly, more elevated in comparison with patients with higher VitD levels. This result may be attributed to sample size.

Elevated neutrophil count predicts ongoing inflammation and decreased lymphocyte count is considered an indicator of poor prognosis. A combination of these two measures, and the derived NLR ratio dNLR, are considered predictive of an inflammatory status. In acute Covid-19 disease, the more severe status is often associated with increased neutrophil cell count and a reduction of lymphocytes. Both CD4 T-helper and CD8 T-cytotoxic lymphocytes may be affected, with a CD4 more severe reduction associated with more severe disease and worst prognosis. In our patients, a moderate correlation was observed between CD4 + /CD8 + ratio, CD8 + count and VitD plasma levels. In addition, a statistically significant difference was detected between patients with low VitD plasma levels in comparison with those with more elevated VitD levels in both CD4 + /CD8 + and CD8 cell count. More elevated CD4/CD8 ratio was detected in patients with low VitD plasma levels. T-lymphocytes are essential in coordinating several immune functions, helping macrophages and B lymphocytes to counteract the development of the disease. The disease-induced loss of lymphocyte activity markedly weakens the immunological responses. Although, the causes of peripheral blood lymphocyte (PBMC) suppression were under investigation, no viral gene expression was detected in PBMC and no COVID-19 virus infection was demonstrated in these cells [32]. Their reduction may be the result of migration/compartmentalization to the site of damaged tissue [33]. In fact, the T CD8 + cell expansion into the lung of mild symptomatic COVID patients, assessed by bronchoalveolar lavage has been described [33].

Categorizing patients as a function of TC Score analyzing lung findings obtained by high resolution computer tomography, we demonstrated that lower plasma levels of Vit D are strictly related with an increase lung involvement characterized by more diffuse ground glass opacities within the lung. This condition reflects more severe disease linked to the redundant and dysfunctional immunological responses described. The same is true describing the sequential organ failure assessment (SOFA) score. It allows us to assess the performance of different organ systems into the body, returning the risk of mortality, based on the relationship between organ failure and mortality. The relationship observed in LIPI and SOFA scores allow us to hypothesize that very low levels of VitD are associated with worst prognosis in COVID-19 patients.

None of the patients studied have had BCG vaccination, patients over the age of 65 had undergone anti-flue vaccination. Although a no-specific protection of vaccination, in particular with BCG, from different viral infections has been reported [34] the protective role of BCG or other vaccination to protect against COVID-19 infection should be interpreted with caution [35]. Furthermore, no significant difference in COVID-19 spread rate was detected between countries with or without current BCG vaccination policy [36].

## Conclusions

Although an inverse correlation between plasma VitD and all causes of mortality in healthy or general medical clinic cohorts has been described in particular in the lowest quantile (0–9 ng/ml), the effect of VitD deficiency in COVID-19 progression or disease severity is far to be assessed. Our data underline a relationship between VitD plasma levels and different serum markers of disease. At the moment it is difficult to argue if VitD supplementation can play a role in fighting the severity of the disease as well as reducing its mortality, but it may be a useful as well as a safe recommendation for almost all patients.

## Data Availability

The data that support the plots within this paper and other finding of this study are available from the corresponding author upon reasonable request.

## Abbreviations

VitD: Vitamin D
CT: Computed tomography
PII: Pulmonary inflammation index
SOFA: Sequential Organ Failure Assessment
LIPI: Lung Immune Prognosis Index
PBMC: Peripheral blood lymphocyte
NLR: Neutrophils and Lymphocytes ratio
dNLR: Derived Neutrophils/ Leucocytes-Neutrophils ratio
TSS: Total severity score
